# Interruption of intermittent hypoxia attenuates the severity of pulmonary fibrosis in male mice

**DOI:** 10.14814/phy2.70335

**Published:** 2025-05-22

**Authors:** Zakaria Maakoul, Celine‐Hivda Yegen, Dominique Marchant, Jean‐Francois Bernaudin, Carole Planès, Nicolas Voituron, Emilie Boncoeur

**Affiliations:** ^1^ Laboratoire Hypoxie & Poumon, UMR INSERM U1272 Université Sorbonne Paris‐Nord Bobigny France; ^2^ Faculté de Médecine Sorbonne Université Paris France; ^3^ Service de Physiologie et d'Explorations Fonctionnelles Hôpital Avicenne, APHP Bobigny France; ^4^ Univ. Grenoble Alpes, INSERM, CEA, UA13 BGE, CNRS, FR2048 ProFI EDyP Team Grenoble France

**Keywords:** intermittent hypoxia, lung damages, macrophages, obstructive sleep apnoea, pulmonary fibrosis

## Abstract

Obstructive sleep apnoea (OSA) leading to chronic intermittent hypoxia (CIH) is a common comorbidity associated with idiopathic pulmonary fibrosis (IPF), a progressive fatal disease characterized by adverse lung remodeling and parenchymal stiffening. In the mouse model of lung fibrosis induced by intra‐tracheal instillation (ITI) of bleomycin, we have previously shown that CIH exacerbates fibrosis, similar to pre‐exposure to ITI. It has been suggested that correction of OSA and CIH with positive airway pressure may have a beneficial effect on the progression of fibrosis in IPF patients. Therefore, we designed this study to determine the benefits of stopping CIH in mice pre‐exposed to CIH for 3 weeks prior to bleomycin ITI. Interruption of CIH exposure was associated with less diffuse lung fibrosis, reduced ratio of damaged area, alveolar destruction, collagen deposition, and fibrosis score. Interruption of CIH exposure significantly reduced macrophage density in fibrotic areas without significant changes in either lymphocyte or neutrophil density. In conclusion, this study demonstrates that early treatment of OSA‐associated CIH can limit or reduce the development of severe forms of pulmonary fibrosis associated with a reduction in macrophage infiltrate and supports the beneficial effect of CIH exposure interruption on ongoing fibrosis severity.

## 1 INTRODUCTION

Idiopathic pulmonary fibrosis (IPF), the archetype of progressive pulmonary fibrosis, affecting mainly men over 50, is characterized by a fatal course of shortness of breath and progressive dyspnoea (Hutchinson et al., 2015) in the absence of truly curative medical treatment (Olson et al., 2018). Although the specific etiology is still unknown, it is widely accepted that the deterioration of the lung architecture and its stiffening would result from repeated microaggressions of the alveolar epithelium and an abnormal repair process (Glass et al., 2022). In addition, many studies have implicated monocyte‐derived macrophages as a key player in the inflammatory process leading to progressive fibrosis (Kim et al., 2025).

In recent years, researchers have highlighted that comorbidities such as OSA may influence the natural history of IPF (Lancaster et al., 2009; Papadogiannis et al., 2021). A study of patients with incident IPF (French cohort COFI) showed that 62% of patients had moderate to severe OSA and 40% had severe OSA (Gille et al., 2017). OSA is characterized by brief and consecutive episodes of hypoxia‐reoxygenation leading to chronic intermittent hypoxia (CIH) due to upper airway obstruction (Dewan et al., 2015). CIH leads to chronic systemic inflammation and has been described to have a significant impact in many pathologies affecting the cardiovascular and nervous systems, metabolism (Gnoni et al., 2022; Sanderson et al., 2021), and the lung (Koritala et al., 2024; Shi et al., 2020). In the lung, exposure to CIH promotes local inflammation and oxidative stress (Koritala et al., 2024; Zhang et al., 2018). Studies in rodent models of bleomycin‐induced pulmonary fibrosis have shown that CIH increases lung oxidative stress in the lung (Lee et al., 2023; Xiong et al., 2021), recruitment of pro‐inflammatory cells and cytokine release (Braun et al., 2018; Gille et al., 2018), and exacerbates lung fibrosis and mortality. Furthermore, the detrimental effects of CIH were exacerbated when initiated before the induction of lung fibrosis (Haine et al., 2021).

Continuous positive airway pressure (CPAP) is an effective treatment for OSA when well tolerated and used correctly (Lazzeri et al., 2024). To date, the impact of OSA treatment on the course of IPF has never been evaluated using a robust methodology. However, preliminary studies suggest that patients with IPF and OSA who are well compliant with CPAP have a higher quality of life and a longer life expectancy than those who are poorly compliant (Mermigkis et al., 2015; Papadogiannis et al., 2021). In the present study, we investigated the effect of early cessation of CIH (at the onset of fibrosis) on both fibrosis severity and the lung inflammatory cell infiltration in a mouse model of bleomycin‐induced pulmonary fibrosis.

## 2 MATERIALS AND METHODS

### 2.1 Animals

The experiments were approved by our ethics committee and by the French Ministry of Higher Education, Research, and Innovation (n° #18309), and performed in accordance with the European Community Council Directive 2010/63/EU on the animals care. Ten male C57BL/6JRj mice (Janvier Labs; 8 weeks old, weight 24 ± 6 g) were used. Mice were housed under standard conditions (12 h/12 h light/dark cycle; food and water ad libitum).

### 2.2 Exposure to chronic intermittent hypoxia and induction of lung fibrosis

**FIGURE 1:**
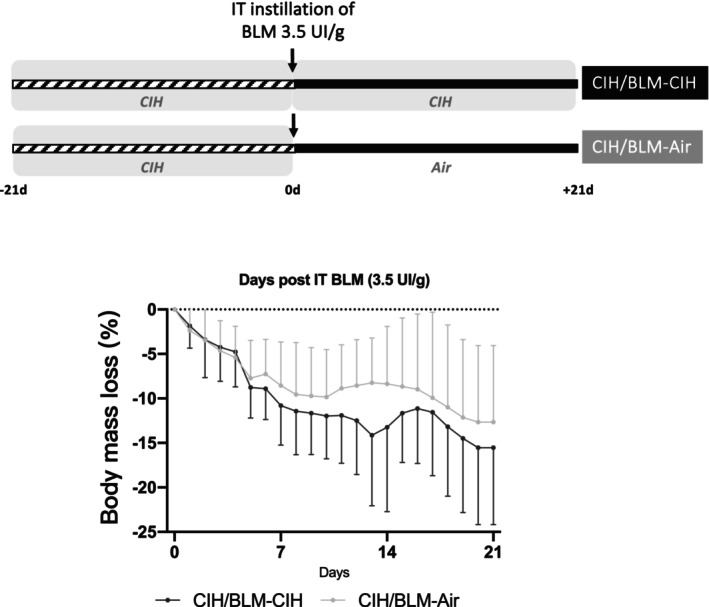
Experimental design and body mass evolution. (a) Mice were exposed to chronic intermittent hypoxia (CIH; 30 cycles/hour, 8 h/day, nadir 7% O_2_) for 21 days. Subsequently, they received an intratracheal instillation (IT) of 3.5 UI/g of bleomycin (BLM) and then half of the animals were left in CIH for another 21 days (CIH/BLM‐CIH; *n* = 5) whereas the other half were left in air (CIH/BLM‐Air; *n* = 5). (b) Body mass was monitored for all mice and was represented by percentage of mass loss from the day of IT instillation of BLM (median ± IR).

Mice were initially exposed to 21 days of CIH (30 cycles/h, 8 h/day, nadir 7% O_2_) as previously described (Gille et al., 2018; Haine et al., 2021) (Figure 1a). After 21 days, pulmonary fibrosis was induced by intra‐tracheal instillation (ITI) of bleomycin (BLM 525709, Bellon–Sanofi, Aventis) at 3.5 IU/g in 150 μL of PBS under anesthesia (Vetflurane, Virbac). Half of the mice were exposed to CIH for a further 21 days (CIH/BLM‐CIH group, *n* = 5), while the other half were exposed to air (CIH/BLM‐Air group, *n* = 5). Body weight and vital signs were monitored daily.

### 2.3 Collection of lung samples

Mice were deeply anesthetized by intraperitoneal injection of ketamine (Virbac, 100 mg/kg)/xylazine (Bayer Healthcare, 20 mg/kg), tracheotomized, and euthanized by abdominal aortic section. Lungs were flushed with PBS via the pulmonary artery. The lobes were insufflated and fixed with 4% paraformaldehyde at a constant pressure (20 cm H_2_O) through the tracheostomy tube. The lungs were removed, post‐fixed in 4% paraformaldehyde (24 h, 4°C), dehydrated, and paraffin‐embedded.

### 2.4 Histological approaches

Lung sections (5 μm) were cleared in xylene, rehydrated, and stained by hematoxylin‐eosin or Masson's trichrome (Yegen et al., 2022). Quantification of affected parenchymal areas and collagen deposition were performed on five microscopic images for each mouse using a Zeiss Axioscope (×400 magnification, ×40 objective). Image J/Fiji toolbox was used for analysis (Ségard et al., 2024). Tissue remodeling was estimated by the ratio of the injured area to the total area. Masson's trichrome was used to quantify the percentage of collagen coverage (aniline bluestained area) using the ColorDeconvolution2 plugin. Quantification of pulmonary fibrosis was assessed using a modified Ashcroft score as previously described (Ashcroft et al., 1988; Yegen et al., 2023) according to five stages from normal to a completely remodeled lung (score 1 to 5). The mean linear intercept method using the grid tool in ImageJ software was used on hematoxylin‐eosin stained sections as a morphometric estimation of alveolar surface destruction, a marker of fibrosis (Xu et al., 2021).

### 2.5 Immunohistochemistry to assess inflammatory cell infiltrates

Antigen retrieval was performed in a boiling citrate buffer. Endogenous peroxidases were blocked with 3% H_2_O_2_, and sections were incubated with 5% horse serum and primary antibodies (24 h, 4°C; Table 1). F4‐80, Ly6G, and CD4 antibodies were used for the identification of macrophages, neutrophils, and T lymphocytes, respectively. An isotopic antibody control was performed. After 24 h, the slides were incubated with biotin‐conjugated secondary antibodies (Dako) and with HRP‐conjugated streptavidin (Vector Lab). DAB (3,3′‐diaminobenzidine) and nuclear fast red (Sigma Aldrich) staining were used to visualize immunolabeling. Semi‐quantification was performed by counting positive cells in three images per mouse at ×400 magnification (objective lens ×40) on Zeiss Axioscope using Histolab image analysis software (V10.1), in an area with large continuous fibrotic masses, with normal architecture without fibrotic burden, and with both architectures.

**TABLE 1:** List of antibodies used for immunohistochemistry.

Antibody	References	Dilution
F4‐80	D2S9R, Cell Signaling Technology	1:250
Ly6G	Ab238132, Abcam	1:500
CD4	D7D2Z, Cell Signaling Technology	1:100
Isotype	Rabbit IgG, Control, Vector Lab (I‐1000)	1:3300
Secondary	Horse Anti‐Rabbit IgG, Biotinylated, Vector Lab (BA‐1100)	1:200

### 2.6 Statistical analysis

Graphical and statistical analyses were performed using GraphPad Prism (GraphPad Software, V9). Normality of data distribution was assessed using the Shapiro–Wilk normality test. Results were expressed as median ± interquartile range (median ± IR). Comparisons between groups were assessed using the Mann–Whitney test, and differences were considered significant when *p* < 0.05.

## 3 RESULTS

During the first 21 days of CIH exposure, prior to the BLM‐ITI, the body mass of the mice was not altered (not shown). After induction of lung fibrosis, the mice lost weight without a significant difference between the two groups (13%–15% decrease observed at D21 in CIH/BLM‐Air group compared to the CIH/BLM‐CIH group) (Figure 1b).

**FIGURE 2:**
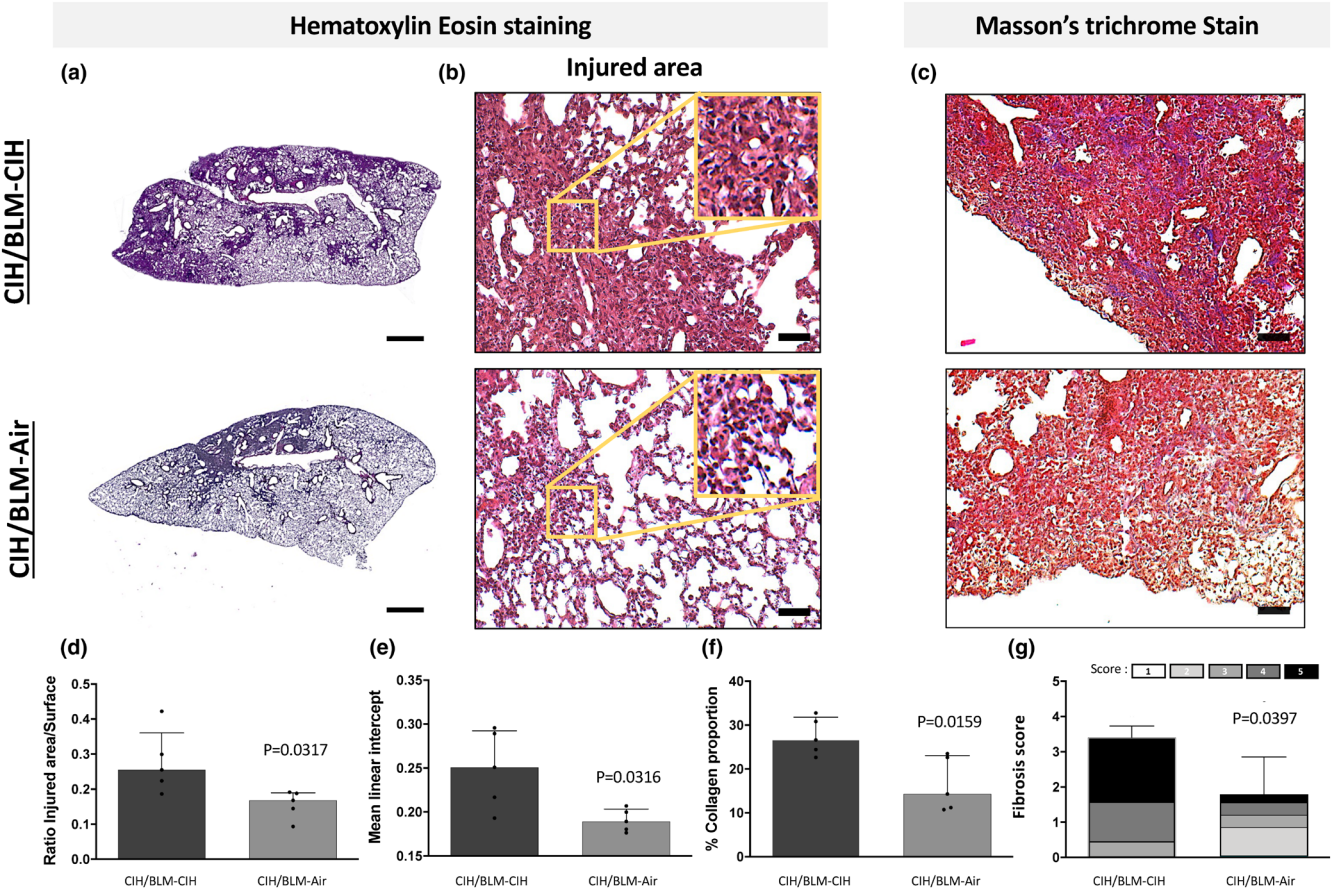
Pulmonary architecture. (a) Cartography of mouse lungs stained with Hematoxylin Eosin. (b) Illustration of injured area (×40 magnification). (c) Histologic illustration of collagen deposition (Masson's Trichrome staining, x40 magnification). (a, b, and c) A representative image of mice lung from the CIH/BLM‐CIH (*n* = 5) and CIH/BLM‐Air (*n* = 5) groups was shown. (d) Quantification of lung injury area reported to the total lung surface, (e) Mean Linear Intercept (MLI) measured on five microscopic images obtained at ×40 magnification of Hematoxylin Eosin‐stained lung biopsy sections, (f) Percentage of collagen occupation on lungs stained with Masson's trichrome of all the mice from the CIH/BLM‐CIH (*n* = 5) and CIH/BLM‐Air (*n* = 5) groups. (g) Histogram bars represent the mean of Aschroft scores with the intra proportion of different fibrotic stages from 1 (normal lung, no fibrotic burden and small fibers) to 5 (completely remodeled lung, large continuous fibrotic masses >50%). Scoring was performed on five images each from CIH/BLM‐CIH (*n* = 5) and CIH/BLM‐Air (*n* = 5) mice.

The CIH/BLM‐CIH group showed changes in the lung architecture (Figure 2a,b), with subpleural and diffuse lesions. Interestingly, the cessation of CIH after BLM‐ITI (CIH/BLM‐Air group) resulted in less significant lung damage compared to the group maintained in CIH exposure (CIH/BLM‐CIH). A significant (*p* < 0.05) 32% reduction in the fibrotic lesions area was observed (Figure 2b,d). Masson's trichrome analysis showed less collagen deposition in the CIH/BLM‐Air group as compared to CIH/BLM‐CIH (Figure 2c), which was confirmed by quantification (Figure 2f). A lower linear intercept indicated less alveolar destruction associated with fibrosis (Figure 2e). The fibrosis score was lower in the CIH/BLM‐Air group with less extensive lesions, mainly in the subpleural area compared to CIH/BLM‐CIH. Furthermore, we also quantified a lower mean lesion severity score from 3.4 ± 1.1 (out of 5) in the CIH/BLM‐CIH group to 1.7 ± 0.74 (out of 5) in the CIH/BLM‐Air group (Figure 2g). In addition, the CIH/BLM‐Air group had fewer areas with severe scores (grades 4 and 5) and the presence of grade 2. In comparison, the CIH/BLM‐CIH group showed only lesions with scores ranging from 3 to 5. Interestingly, despite the reduction in fibrosis score, no area without fibrotic burden has been observed (score of 1) in either group (Figure 2g).

**FIGURE 3:**
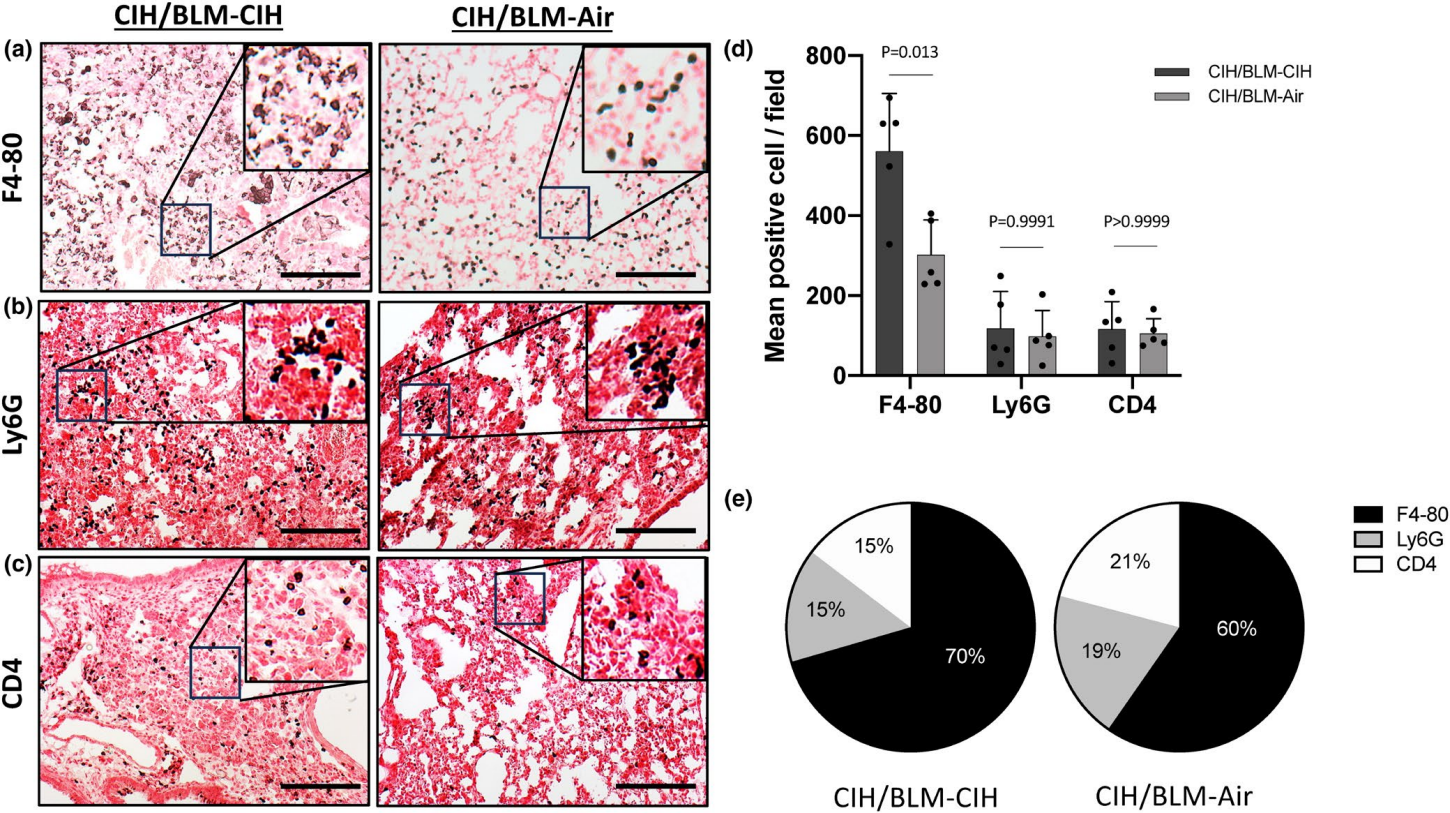
Pulmonary inflammatory response. (a, b, and c) Analysis of differential inflammatory cells by immunohistochemistry for total F4‐80 (a), Ly6G (b), and CD4 positive cells (c) performed on 5 μm lung biopsy sections. A representative image of mice from the CIH/BLM‐CIH (*n* = 5) and CIH/BLM‐Air (*n* = 5) groups is shown. (d) Semi‐quantification of F4‐80, Ly6G, and CD4 positive cells counted in lung sections from CIH/BLM‐CIH (*n* = 5) and CIH/BLM‐Air (*n* = 5) mice. (e) Proportions of macrophages (F4‐80), lymphocytes (CD4), and neutrophils (Ly6G) in the CIH/BLM‐CIH (*n* = 5) and CIH/BLM‐Air (*n* = 5) groups.

Regarding lung inflammation, the CIH/BLM‐Air group showed fewer inflammatory cells (Figure 3), due to a 50% reduction in the number of macrophages (F480) (Figure 3a,d) as compared to the CIH/BLM‐CIH group (302.2 ± 86.7 vs. 561.2 ± 143.9 cells respectively, *p* < 0.05). In contrast, there was no difference in the number of T lymphocytes (CD4) and neutrophils (Ly6G) (Figure 3b,c). This led to a change in the proportion of each cell type (Figure 3e). F4‐80 positive cells, which represented 70% of the total population analyzed in the CIH/BLM‐CIH group, represented only 60% in the CIH/BLM‐Air group. The proportions of CD4 and Ly6G‐positive cells increased by approximately 5% (from 15% to 21% and from 15% to 19%, respectively) (Figure 3e).

## 4 DISCUSSION

We have previously shown that CIH exacerbates BLM‐induced pulmonary fibrosis in mice (Gille et al., 2018) and that this worsening effect was strengthened when CIH was initiated before the induction of lung fibrosis and continued thereafter (Haine et al., 2021). Here, we propose to document the severity and evolution of lung fibrosis when CIH is interrupted at the onset of lung fibrosis. Our results showed that this interruption with reintroduction of air breathing significantly reduced the severity of lung injury and inflammatory status.

Exposure to CIH at the onset of fibrosis induction in the BLM‐mouse model has previously been shown to induce a higher mortality, associated with a more severe fibrosis as assessed by lung collagen content and histological characterization (Gille et al., 2018; Haine et al., 2021). In addition, neutrophilic alveolitis and increased oxidative/nitrosative stress were observed in the first week after BLM instillation in the lungs of mice exposed to CIH, followed by pulmonary oedema and increased lung cell apoptosis (Gille et al., 2018). Furthermore, when CIH was applied 2 weeks before the onset of fibrosis, induction of pro‐fibrotic markers, increased tissue injury, and collagen deposition were observed (Haine et al., 2021). This demonstrates the detrimental effect of CIH exposure on the induction and severity of fibrosis whether it is applied before or at the same time as BLM instillation. Our study showed deep lung remodeling in the CIH/BLM‐CIH group with reduced mean air space area, increased collagen deposition, and a notable proportion of high Ashcroft scores (more than 50% of slides at level 5). Finally, we showed a high proportion of macrophage population in the lung (70%), within the range of values classically found in the literature (Kim et al., 2010).

When CIH was stopped at the onset of pulmonary fibrosis induction (i.e., at the time of BLM instillation), we observed an overall less severe form of lung fibrosis, less diffuse lung fibrosis with a reduced ratio of damaged area, reduced collagen deposition and fibrosis score, and a larger air‐filled area. Several hypotheses can be put forward to explain these observations. The first hypothesis which was also the subject of this study is a global change in the inflammatory state as a consequence of the interruption of CIH. Indeed, we have shown that cessation of CIH specifically reduces the number of macrophages in the affected areas, while other inflammatory cells remain unchanged. Given the critical role of macrophage accumulation in lung fibrogenesis (Fabre et al., 2023) and the link between lung macrophage dynamics and inflammation/fibrosis (Aisanjiang et al., 2024), a reduction in pro‐inflammatory macrophages may lead to a less severe form of fibrosis. This explanation is consistent with the observation that patients treated with CPAP have reduced the inflammatory cytokine release (Mahboub et al., 2022). However, to our knowledge, the effect of CPAP treatment on the lung inflammatory status of IPF patients with OSA has never been documented.

The reduction in lung lesion severity observed after cessation of CIH may be due to a reduction in M2‐polarized macrophages and released pro‐fibrosing factors (Braun et al., 2018; Kim et al., 2025), as a consequence of the overall reduction in the macrophage population. It would be interesting to evaluate the modulation of these specific molecular markers secreted by macrophages (i.e, TGFβ, IL13, or VEGF) with and without the interruption of CIH in the broncho‐alveolar lavage of mice or the activation of downstream signaling pathways within the lung tissue. An alternative explanation would be a reduction in oxidative stress and reactive oxygen species, which could limit epithelial cell injury and apoptosis (Gille et al., 2018). This hypothesis remains to be explored. Finally, the beneficial impact of cessation of CIH on the severity of lung fibrosis at the indicated time point could also be due to the repair process activation. This point and the signaling pathways involved should be further investigated.

In conclusion, this study highlights the beneficial effect of cessation of CIH on the severity of the ongoing fibrosis and provides a proof of concept that early treatment of OSA‐associated CIH limits the development of severe progressive forms of lung fibrosis.

## AUTHOR CONTRIBUTIONS

NV and EB designed the experiments. ZM, C‐HY, DM, JF‐B, NV, and EB performed and analyzed the experiments. NV and EB wrote the manuscript. ZM, C‐HY, DM, JF‐B, CP, NV, and EB discussed the data and revised the manuscript. All authors approved the submitted version.

## FUNDING INFORMATION

“Institut Fédératif de Recherche Biomédicale” programs of the USPN (NV‐2020) and “Legs Poix” grant (NV‐2018).

## CONFLICT OF INTEREST STATEMENT

No conflicts of interest.

## ETHICS STATEMENT

The experiments were approved by our ethics committee and by the French Ministry of Higher Education, Research, and Innovation (n° #18309), and performed in accordance with the European Community Council Directive 2010/63/EU on the animals care.

## Data Availability

Data will be made available on request.
